# Case report: a case of lung squamous cell carcinoma with a novel FGFR3-IER5L fusion mutation responding to anlotinib

**DOI:** 10.3389/fonc.2024.1391349

**Published:** 2024-10-03

**Authors:** Xiaoting Chen, Wen Zhao, Hejiang Yu, Shuang Wang, Chengjun Wang, Yanan Song, Xue Meng, Jisheng Li

**Affiliations:** ^1^ Department of Oncology, The Second Affiliated Hospital of Shandong First Medical University, Taian, Shandong, China; ^2^ Department of Medical Oncology, Qilu Hospital, Cheeloo College of Medicine, Shandong University, Jinan, Shandong, China; ^3^ Department of Oncology, Yunyang County People’s Hospital, Chongqing, China; ^4^ Department of Radiation Oncology, Shandong Cancer Hospital and Institute, Shandong First Medical University and Shandong Academy of Medical Sciences, Jinan, Shandong, China

**Keywords:** lung squamous cell carcinoma, FGFR3-IER5L fusion, anlotinib, multi-target tyrosine kinase inhibitor, driver gene mutation

## Abstract

Lung squamous cell carcinoma (LUSC) is the second most common pathological type of non-small cell lung cancer (NSCLC). However, compared with lung adenocarcinoma (LUAD), the incidence of driver gene mutations in LUSC is relatively lower and treatment options for LUSC patients are very limited. We described a LUSC patient with a novel FGFR3-IER5L fusion revealed by next generation sequencing in this report. The patient refused surgery, radiotherapy or chemotherapy and received anlotinib treatment. Anlotinib is a small molecular multi-target tyrosine kinase inhibitor, which can inhibit the activity of kinases including vascular endothelial growth factor receptor 2/3 (VEGFR2/3), fibroblast growth factor receptor 1-4 (FGFR1-4), platelet-derived growth factor receptor α/β (PDGFRα/β), and c-Kit. The patient achieved partial response and the progression-free survival was 3.8 months.

## Introduction

Lung cancer ranks first in contributing to cancer-related death worldwide. Non-small cell lung cancer (NSCLC) accounts for about 85% of all lung cancer cases, of which 25%-30% are lung squamous cell carcinoma (LUSC) (1, 2). Clinically, LUSC often grows along the proximal bronchi and invades large blood vessels. And most of the patients are elderly male with a smoking history, accompanied with cardiopulmonary complications (3). Compared with lung adenocarcinoma (LUAD), reduced prevalence of driver mutations was observed in advanced LUSC patients and a limited targeted drugs were available (4, 5). The treatment of LUSC is faced with great challenges because of its unique clinical and biological characteristics. Chemotherapy and immunotherapy are the main medical treatment strategies.

Whether to conduct routine molecular testing in LUSC has been debated for years. Considering the low prevalence of actionable alterations for LUSC patients, molecular testing was only recommended in never smokers or small biopsy specimens or mixed histology and only included epidermal growth factor receptor (EGFR) mutation and anaplastic lymphoma kinase (ALK) mutation testing before 2021. As utilization of next-generation sequencing (NGS) and liquid biopsy increases, this premise is reevaluated. A study showed that in a 467 LUSC patient cohort, the proportion of somatic alterations with therapeutic relevance was as high as 10.5%, including in EGFR (2.8%), ALK/ROS1 (1.3%), BRAF (1.5%), and MET amplification or exon 14 skipping (5.1%) (6). In another cohort, 172 LUSC patients were included and 130 patients had evaluable NGS results, of which 49 (38%) had at least 1 alteration qualifying for an approved therapy or other clinical trial (7). Based on results of the two researches, the National Comprehensive Cancer Network (NCCN) NSCLC guidelines recommend routine molecular testing in LUSC patients and broad molecular profiling not limited to EGFR and ALK mutations (8).

Fibroblast growth factor receptor (FGFR) family consists of four subtypes of transmembrane tyrosine kinase receptors and they play an important role in stimulating cell proliferation, differentiation and angiogenesis by activating mitogen-activated protein kinase (MAPK) signaling, and PI3K/Akt signaling (9). FGFR family has been identified as a novel and potential therapeutic target in NSCLC patients. Previous research showed that FGFR aberrations, including point mutations, gene fusions and amplification, were detected in about 1.9% of NSCLC patients (10). Besides, FGFR fusions were more frequently observed in LUSC (3.5%) than LUAD (0.6%) (11). FGFR3 fusion has recently been identified as a driver mutation in LUSC (12, 13), glioblastoma (14) and bladder cancer (15). However, most of the researches focus on FGFR3-TACC3 gene fusions. To our knowledge, FGFR3-IER5L fusion has not been reported in any cancer types by now.

Anlotinib is a novel, orally administered, small molecule multi-target tyrosine kinase inhibitor (TKI). It is originally designed to target vascular endothelial growth factor receptor (VEGFR2/3), fibroblast growth factor receptor (FGFR1-4), platelet-derived growth factor receptor (PDGFRα/β), and c-Kit; thus, it has broad inhibitory effects on tumor angiogenesis and growth (16). Anlotinib has been approved as third or further-line treatment for NSCLC patients by the National Medical Products Administration (NMPA) of China (17). Case reports showed that anlotinib was effective in treating patients with FGFR fusions, including FGFR3-TACC3 and FGFR2-ERC1 fusion (18, 19). In this study, we presented a LUSC case with a newly found FGFR-IER5L fusion. And this patient showed a partial response to anlotinib single agent treatment.

## Case presentation

A 76-year-old man was admitted to hospital owing to cough for several months. He had a history of smoking for more than 30 years with 20 cigarettes per day, and quitted it 2 years ago. Family history of genetic disease or tumor was denied. Routine blood, fecal, and urine tests returned normal results. Comprehensive evaluations of cardiac, hepatic, renal, pulmonary, coagulation, and electrolyte functions showed no significant abnormalities. Tumor markers, including CEA, Cyfra21-1, and SCC, were within normal ranges. Physical examination indicated a Karnofsky Performance Status (KPS) of 80%, with less than 5% weight loss over three months. No enlargement of superficial lymph nodes was detected. Thoracic and pulmonary examinations were unremarkable, and no other notable abnormalities were observed. Contrasted CT scan revealed a big mass in the lower lobe of the right lung, measuring 5cm×3cm in size with uneven enhancement. There was no evidence of mediastinal lymph nodes enlargement. PET/CT and cranial MRI revealed no distant metastasis. Biopsy was conducted and it showed LUSC. According to the AJCC Cancer Staging Manual (8th edition) for lung cancer, the clinical staging was T2bN0M0 IIA. The patient and family members had been fully informed of with recommended treatment modalities especially the radical local treatment opportunity including surgery and radiotherapy. However, after careful consideration, they refused surgery, radiotherapy as well as medical treatment including chemotherapy and immune checkpoint inhibitor treatment, due to serious concerns about potential risks and adverse events.

**Figure 1:**
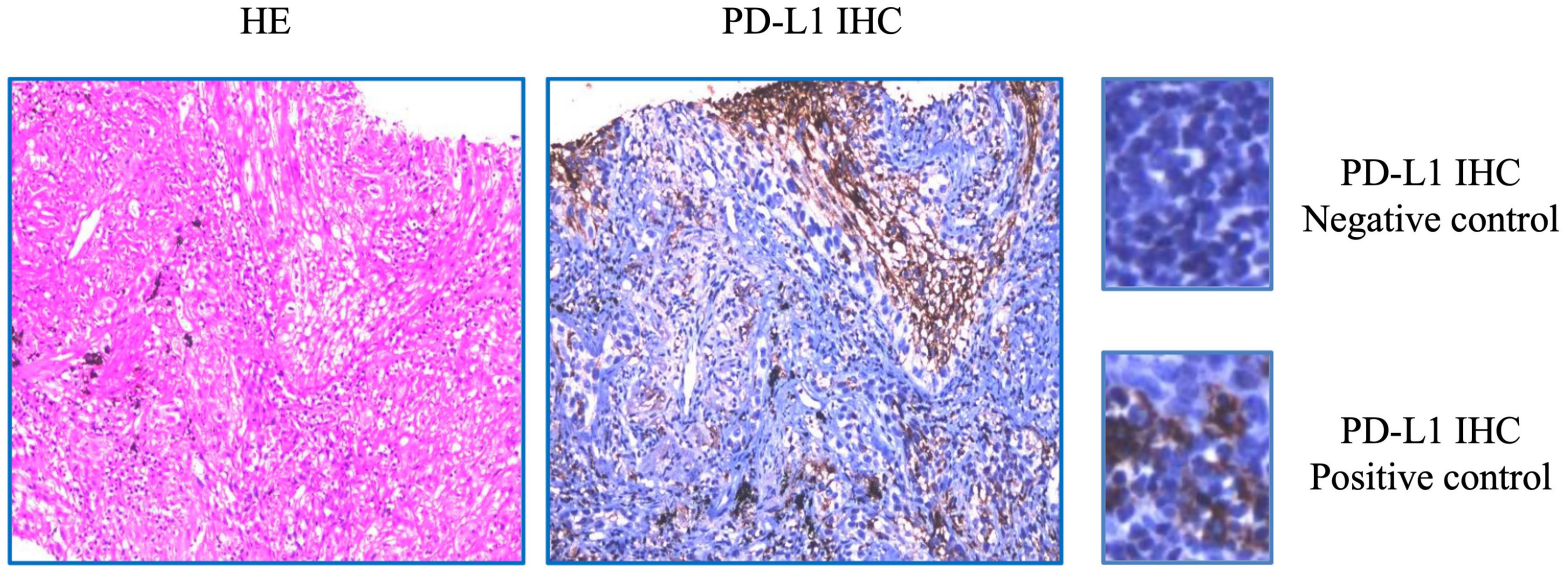
Expression of PD-L1 protein detected by PD-L1 22C3 antibody. immunohistochemistry (IHC).

**Figure 2:**
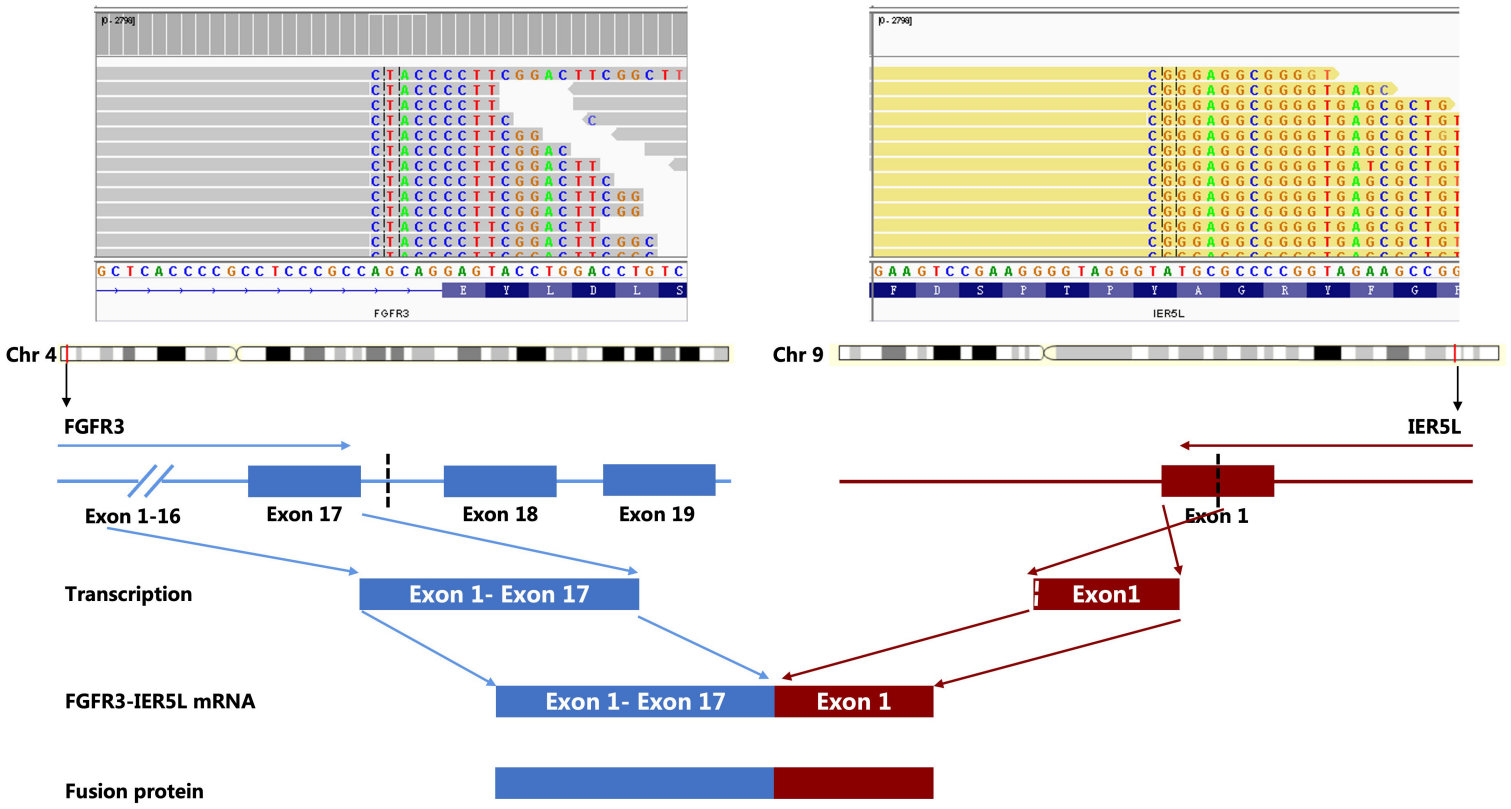
Structure of FGFR3-IRE5L fusion identified by next-generation sequencing.

NGS analysis revealed a novel FGFR3-IER5L fusion mutation with mutation allele frequency (MAF) of 12.5%, a NF1 shift mutation (MAF=13.4%), a TP53 nonsense mutation (MAF=11.1%), a CSF1R splicing mutation (MAF=13.7%) and a missense mutation in ROS1 exon 34 (MAF=15.4%) (**Table 1**). It also showed a low tumor mutation burden (TMB) of 5.3 mut/Mb and microsatellite stability (MSS). Tumor proportion score (TPS) of programmed death-ligand 1 (PD-L1) was 3% detected by 22C3 antibody (**Figure 1**). **Figure 2** showed the genetic structural details of FGFR-IER5L fusion. The FGFR-IER5L fusion protein may stimulate FGFR3 kinase activity and downstream signaling pathways, including MAPK signaling and PI3K/AKT signaling, leading to tumor development. At present, there is no standard treatment strategy for LUSC patients with FGFR3 fusion mutation and whether these patients will benefit from current FGFR target therapy remains unknown.

**Table 1:** Tumor-specific mutations in this patient detected by next-generation sequencing.

Gene	Variation type	Mutation site	Mutation allele frequency
**FGFR3**	Fusion mutation	FGFR3: exon17~IER5L: exon1	12.5%
**NF1**	Frameshift mutation	c.755_756insA (p. V253Gfs*6)	13.4%
**TP53**	Nonsense mutation	c.1005_1006delinsAT (p.E336*)	11.1%
**CSF1R**	Splicing mutation	c.1083-1G>A	13.7%
**ROS1**	Missense mutation	c.5603T>C (p.V1868A)	15.4%

**Figure 3:**
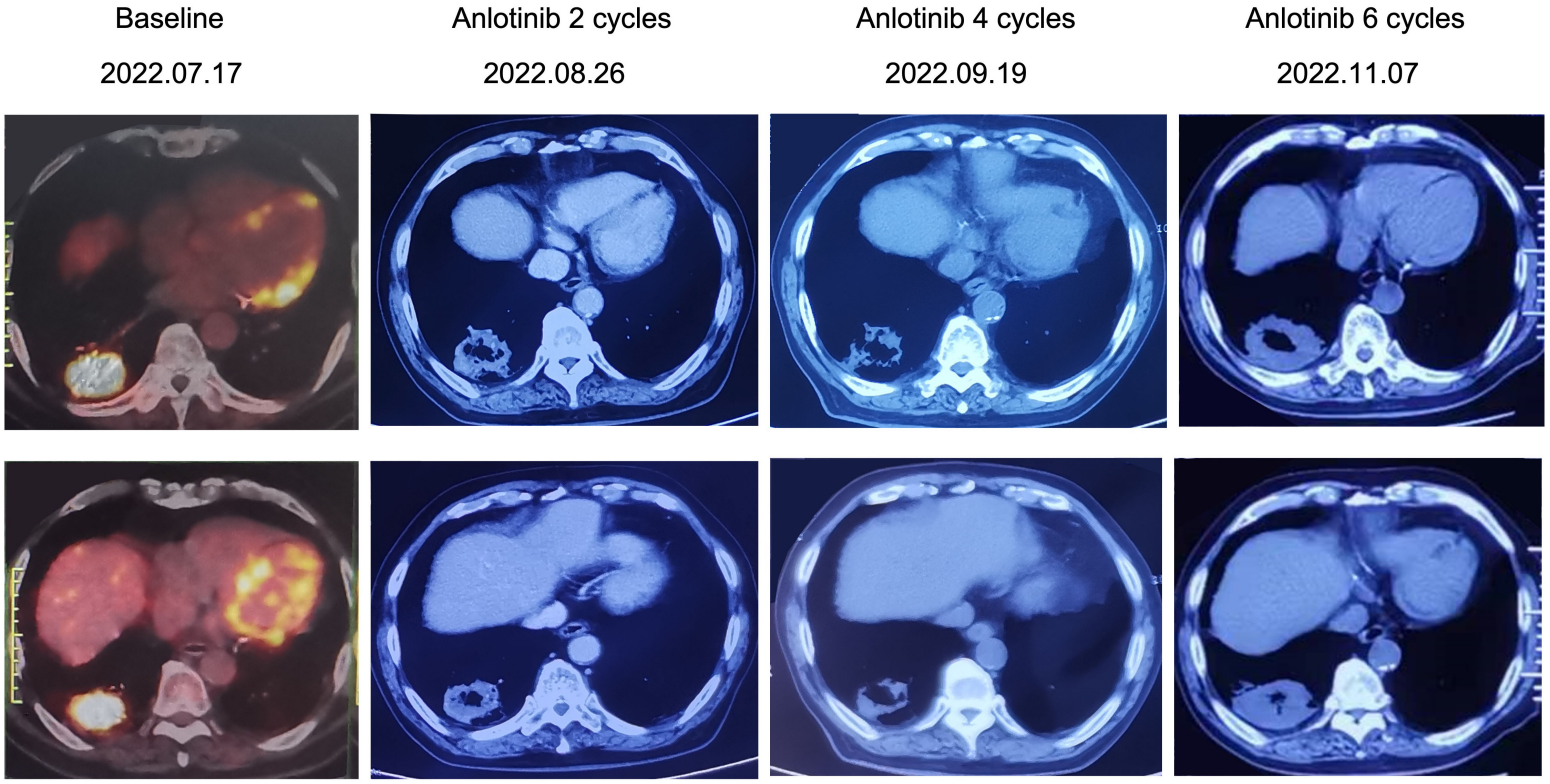
Timeline of anlotinib treatment and radiographic responses.

**Figure 4:**
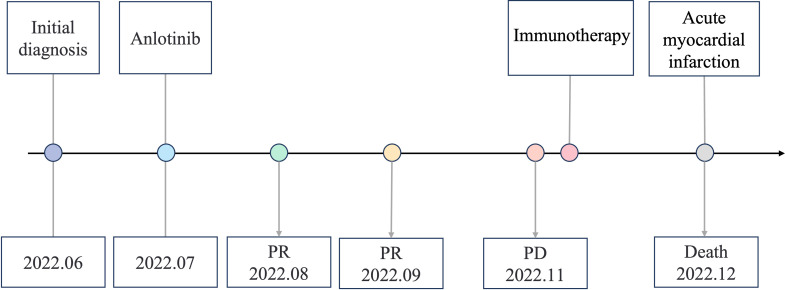
Timeline of the clinical diagnosis and treatment.

A variety of FGFR-targeted agents have been developed, including pan-FGFR inhibitors such as erdafitinib and futibatinib, and FGFR1/2/3 inhibitors like infigratinib and pemigatinib. Some of these agents have been approved and are in clinical use. However, there is no established treatment strategy for patients with LUSC harboring FGFR3 fusion mutations, and it remains uncertain whether these patients will benefit from current FGFR-targeted therapies. Due to considerations of drug indications, accessibility, and cost, this patient declined treatment with the currently available FGFR inhibitors. Anlotinib is a novel small molecule tyrosine kinase inhibitor and effectively inhibits the activity of multiple targets, including FGFR3. A few case reports showed that it is effective in patients with FGFR2/3 fusion. And anlotinib has been approved as a third-line treatment choice for advanced NSCLC patients. The patient was administered with anlotinib 12 mg p.o. every morning (days 1-14, with a 21-day cycle). After anlotinib treatment for 2 cycles, the patient achieved a partial response, with an obvious cavity inside the tumor. The adverse effect (AE) was only mild hypertension and was well controlled. We didn’t observe any serious AEs occurred during anlotinib treatment in this case. However, CT scan showed disease progression after 6 cycles of anlotinib treatment and the progression-free survival (PFS) was 3.8 months (**Figure 3**). After progression, the patient's family still refused radiotherapy and received one cycle of immune checkpoint inhibitor in local hospital. On December 2022, the patient passed away due to acute myocardial infarction. The timeline of the clinical diagnosis and treatment of this patient is summarized in **Figure 4**.

## Discussion

To our knowledge, this is the first case report describing a novel FGFR3-IER5L fusion in a LUSC patient and the patient showed partial response to anlotinib, a small molecule multi-target tyrosine kinase inhibitor targeting VEGFR, FGFR and PDGFR.

Routine molecular testing was not recommended in LUSC until 2021 because of the low driver gene mutation rate shown in small sample studies. Two large cohort studies showed that in LUSC patients, somatic alteration rate was as high as 10.5%. Besides, not only EGFR and ALK, but also ROS1, BRAF, MET, PIK3CA, FGFR family, and TSC1/2 mutations were detected (6, 7). Due to the large number of LUSC patients, identifying even a small subset of patients with gene abnormalities potentially responsive to targeted therapy holds great clinical significance. Increased insight into the mutational landscape has contributed a lot to the development of effective targeted therapies for LUSC patients. Recently, TORC1/2 inhibitor sapanisertib (TAK-228) exhibited promising efficacy in NRF2-mutated advanced LUSC with an objective remission rate (ORR) of 25% and a median PFS of 8.9 months (20). Based on clinical trial data, the NCCN NSCLC guideline recommends molecular testing, especially broader molecular profiling to identify common and other rare driver mutations for which targeted therapies may be useful in LUSC (8). Notably, molecular testing should be done in all patients with metastatic LUSC, and not just those with certain characteristics, such as never-smoking, small biopsy specimens, and mixed histology.

FGFR is a transmembrane receptor tyrosine kinase, including FGFR1-4. The FGFR family has been identified as a novel potential therapeutic target in diverse cancer types (12–15). In physiological state, FGFR is phosphorylated and dimerized by binding to ligand fibroblast growth factor (FGF), which activates downstream signaling pathways and actively participates in cell proliferation, differentiation, survival, migration, angiogenesis and DNA damage repair (9). FGFR is the most frequently mutated tyrosine kinase family gene in LUSC, accounting for about 12%-20% cases (9). FGFR gene alteration mainly include gene amplification, point mutations and gene fusions. FGFR1-4 fusions have been reported in LUSC (11, 21), of which FGFR1, FGFR2, FGFR3 and FGFR4 accounts for ~ 18%, 2.5–4.7%, 0–9%, and 5.3%, respectively (22). Specifically, the mutation incidence of FGFR3 fusions is about 6.8% (9). Different from EGFR mutations, FGFR fusions are more common in smoking people than in former-smokers and never-smokers (11). Immediate early response 5-like (IER5L) is a member of the immediate early response (IER) family, including IER2 and IER5. The IER family regulates the phosphorylation status of various kinases, including heat shock factor 1 (HSF1), ribosomal protein S6 kinase (S6K), and cell division cycle 25A (CDC25A) (23, 24). In this case, we detected a FGFR3-IER5L fusion for the first time. The mechanism of constitutive activation and associated signaling pathways are still unclear. The majority of FGFR fusion occurring in-frame result in a functional chimeric protein (25). As other FGFR fusions reported in solid malignancies, FGFR3 C terminus is involved in FGFR3-IER5L fusion classified as type II fusions (26). Usually, type II fusions have a loss of the phospholipase-C-binding tyrosine and lead to regulated signal transduction (27). However, whether FGFR3-IER5L fusion leads to ligand-independent receptor dimerization or increased kinase activity in the fusion protein remains to be investigated.

In view of the contribution of aberrant FGFR signaling to tumorigenesis, several target agents have been developed. Currently, four FGFR inhibitors have been approved internationally for the treatment of advanced solid tumors with distinct FGFR gene variants. These include erdafitinib, futibatinib, pemigatinib, and infigratinib. Erdafitinib has been approved for patients with urothelial carcinoma harboring FGFR2 or FGFR3 mutations. Notably, the THOR trial demonstrated that patients treated with erdafitinib achieved a median overall survival (OS) of 12.1 months, compared to 7.8 months for those receiving chemotherapy. This significant improvement highlights erdafitinib’s clinical efficacy in this patient population (28). Futibatinib and pemigatinib have received approval for the treatment of cholangiocarcinoma patients with FGFR2 fusions or rearrangements. Pemigatinib, evaluated in the FIGHT-202 trial, exhibited a 36% overall response rate in patients with cholangiocarcinoma harboring FGFR2 fusions or rearrangements (29). Furthermore, pemigatinib’s efficacy has been demonstrated across various tumor types. The FIGHT-207 study assessed pemigatinib’s effectiveness and safety in previously treated advanced, metastatic, or unresectable solid tumors, including breast, bile duct, central nervous system, gynecologic, non-small cell lung, pancreatic, and urothelial/bladder cancers with FGFR mutations, fusions, or rearrangements. This study underscores pemigatinib’s broad anti-tumor activity across multiple cancer types (30). Infigratinib was approved for cholangiocarcinoma in 2021 but has since been withdrawn from the U.S. market. Nonetheless, its initial approval indicates its potential efficacy in FGFR-driven cancers.

At present, there are still no FGFR-targeted therapies approved for the treatment of LUSC. Case reports have demonstrated that FGFR inhibitors can be effective in treating NSCLC patients with FGFR aberrations (31). The RAGNAR trial evaluated the efficacy of the pan-FGFR inhibitor erdafitinib in adults with NSCLC who had pre-specified FGFR alterations. The ORR was 26% (95% CI: 10-48), with 21% in LUSC and 33% (3/9) in non-LUSC patients. Gene alterations included FGFR2/FGFR3 mutations and fusions. The median duration of response, PFS, and OS were 4.6 months, 4.1 months, and 10.5 months, respectively (32).

Anlotinib is a small molecular multi-target TKI, effectively inhibiting the activity of kinases including VEGFR, FGFR, PDGFR, and stem cell growth factor receptor. Based on the good efficacy and safety of anlotinib in patients with progressive or recurrent NSCLC, NMPA approved it for third-line and further treatment in patients with progressive or recurrent NSCLC. Anlotinib has also been reported useful in patients carrying FGFR fusions. An anaplastic astrocytoma patient with FGFR3-TACC3 fusion experienced tumor relapse from local therapy and systemic therapy of temozolomide and bevacizumab. At a later-line therapy, she achieved more than 17 months of PFS from temozolomide and anlotinib therapy (18). Another case showed a female patient with LUAD who underwent right upper lobectomy and adjuvant chemotherapy. Thirteen months later, the disease recurred and she had primary resistance to chemotherapy and immune checkpoint inhibitor with a PFS of only 2 months. A FGFR2-ERC1 fusion was confirmed by NGS and anlotinib was administered. The PFS was 8.0 months and she was still at follow-up when reported (19). The mechanism of action of anlotinib on FGFR3 fusion gene-positive tumors may include the following. Firstly, anlotinib targets FGFR3. By inhibiting FGFR3, anlotinib blocks downstream signaling pathways, such as the RAS/RAF/MEK/ERK and PI3K/AKT pathways, which are crucial for tumor growth and survival. This inhibition reduces the activation of these pathways, thereby inhibiting cell proliferation, survival, and angiogenesis. Secondly, in addition to FGFR3, anlotinib also inhibits other receptor tyrosine kinases such as VEGFR and PDGFR. This broader inhibition can provide a more comprehensive approach to tumor growth suppression compared to agents that specifically target FGFR alone, potentially leading to improved efficacy.

In this case, although diagnosed at an early stage, the patient refused radiotherapy, chemotherapy or surgery. He achieved a partial response on anlotinib treatment, but the PFS is shorter than 4 months. It may be explained by acquired resistance to anlotinib. NGS also showed a TP53 nonsense mutation in this case. A previous study reported TP53 negatively correlated with efficacy of EGFR-TKI (33). And in the clinic genomic analysis of FIGHT-02 (34), it showed that TP53 mutations almost exclusively co-existed with FGFR mutation. Patients carrying both of FGFR and TP53 mutation exhibited worse response to FGFR-TKI pemigatinib. In this case, the relatively shorter duration of response may be explained by the TP53 co-mutation. However, the molecular mechanism of different FGFR3 fusions is still unclear and needs to be clarified.

In previous studies, the most common grade 3 or higher adverse events during anlotinib treatment in advanced non-small cell lung cancer were hypertension, triglyceride elevation, hand and foot skin reaction, hyponatremia and lipase elevation (17, 35). Anlotinib is a tyrosine kinase inhibitor targeting multiple receptors, particularly VEGFR. The VEGF signaling pathway regulates various endothelial cell functions through complex interactions with multiple signaling pathways. Consequently, inhibition of the VEGF signaling pathway can impair neovascularization, disrupt platelet-endothelial cell interactions, and obstruct both the coagulation and platelet activation systems, thus reducing wound healing ability and increasing the risk of bleeding. To mitigate the risk of bleeding, the Chinese Society of Clinical Oncology (CSCO) recommends anlotinib primarily for the treatment of peripheral lung squamous cell carcinoma. It is worth noting that although in the ALTER0303 study and ALTER1202 study, anlotinib did not significantly increase the incidence of grade 3 or larger hemoptysis in patients with lung cancer (17, 36). In this case, the lesion was located in the lower lobe of the right lung, where the bleeding risk was considered low. The AE observed was only mild hypertension and was well controlled. We didn’t observe any triglyceride elevation, hand and foot skin reaction, lipase elevation or hemoptysis.

In a word, herein we report a LUSC patient with a novel FGFR3-IER5L fusion, and anlotinib showed partial response in this case. The role and significance of FGFR gene fusion mutations in NSCLC need more investigation for novel treatment strategies.

## Data Availability

The original contributions presented in the study are included in the article/supplementary material. Further inquiries can be directed to the corresponding authors.
